# Neuroaxial anesthesia in a patient with progressive systemic sclerosis : case presentation and review of the literature on systemic sclerosis

**DOI:** 10.1186/1471-2253-6-11

**Published:** 2006-10-17

**Authors:** Gülcan Erk, Vildan taşpınar, Ferah Dönmez, Dilşen örnek

**Affiliations:** 1 Department of Anesthesiology and Reanimation, Ankara Numune Training and Research State Hospital, Ankara, Turkey

## Abstract

**Background:**

Systemic sclerosis (SSc), a progressive disease characterized by excessive accumulation of connective tissue components. Although most patients have long survival, some of them progress rapidly to death. Pulmonary system involvement and pulmonary hypertension are the most frequent cause of death. When the patient with SSc is to be operated, the anesthetic procedure could be a serious problem. In this article, we report a combined spinal – epidural technique in a patient with progressive SSc and the anesthetic considerations that could be recommended for these patients.

**Case presentation:**

A 68-year-old woman who had a history of progressive systemic sclerosis, pulmonary fibrosis, kyphoscoliosis and decreased oral apertura underwent total hip arthroplasty. This operation was performed successfully under combined spinal epidural anesthesia.

**Conclusion:**

Systemic sclerosis is a complex disease that involves multiple organ systems. Every aspects of anesthetic care may be altered or hindered by the pathogenesis of disease. Although the choice of regional or general anesthesia is unclear, to choose combined spinal epidural anesthesia may be useful.

## Background

Systemic sclerosis, also termed 'scleroderma' is a multsystem- connective tissue disease, characterized by excessive fibrosis, vascular abnormalities, and immune dysfunction. A relatively rare syndrome, scleroderma has an annual incidence of 1 or 2 cases per 100 000 and 3 times more frequent in women than men. The usual onset is between 30 to 50 years of age. Muscles, bones, mucous membranes, heart, lungs, intestinal tract and other internal organs may be involved [1]. Kidney, lung and heart involvement is associated with poor prognosis; moreover visceral involvement and immunological findings have been considered as prognostic factors by some authors [2]. Sixty percent of deaths are secondary to heart or lung involvement and (or) to pulmonary hypertension[3].

It is reported that, anesthesiologists managing patients with SSc intraoperatively must have experience and background about the pathogenesis, clinical manifestations, systemic involvement and anesthetic considerations. One should never forget that every aspects of anesthetic care may be altered or hindered by the pathogenesis of this disease [4].

In this report we present a very rare case of combined spinal-epidural anesthesia in a patient with SSc whose pulmonary function tests were decreased with low exercise capacity.

## Case presentation

A 68 year-old woman, 150-cm in height, weighing 45-kg with traumatic left femur fracture admitted to the hospital for total hip arthroplasty. She had been diagnosed of SSc ten years ago. Before surgery she was receiving deflazacort 13.5 mg/day every other day, pentoxifyllin 400 mg/day, acetylsalicylic acid 300 mg/day, cyclophosphamide 50 mg/day, colchicine 1 mg/day and risedronate sodium 35 mg/week. Deflazacort, acetylsalicylic acid, cyclophosphamide and colchicine were quited preoperatively.

On her physical examination, she has waxy, smooth and tight skin, decreased oral aperture (3 cm) and thyromental distance was 6 cm, the patient was almost bedridden secondary to severe flexion contractures of all four extremities. She had borderline hypertension (150/90 mmHg). She had chronic cough with bilaterally reduced lung sounds. Inspection of her back revealed kyphoscoliosis. Because of severe dyspne, she had echocardiyagram, the result was compatible with normal left ventricular size and contractility, minimal right atrial enlargement, minimal dilated hypo kinetic right ventricle. Bibasal pulmonary fibrosis was detected on her chest radiogram. The arterial blood gases and pulmonary function tests result were as fallow; fraction of inspired oxygen 0.21, PaO_2 _: 88.6 mmHg, PaCO_2 _: 18.8 mmHg, and pH: 7,51; forced expiratory volume in 1^st ^second (FEV_1_):1,5 L, forced vital capacity (FVC): 1.9 L, total lung capacity 2,95 L. The liver and renal function tests were normal. The complete blood caunt was as follows. Hemoglobin:10.5 g/dl, platelet (plt) : 202 K/uL, protrombine time (PT):11.88 s (range:11.5–15 s) and activated partial thromboplastin time (aPTT): 23.22s (range: 20–33 s).

The patient received 150 mg of ranitidine and 10 mg of metoclopramide thirty minutes before the regional block. In the operation room, arterial blood pressure (BP) was 153/92 mmHg, and her heart rate (HR)was 95 bpm. A 16 gauge venflon catheter was inserted into a vein in the dorsum of right hand, and 500 ml of 0.9 % NaCl solution was infused over the next 20 min. Standard monitoring was used throughout the procedure, including continuous ECG (Lead II), heart rate, non-invasive arterial blood pressure measured in every five minutes and continuous pulse oximetry. A senior experienced anesthesiologist performed the combined spinal- epidural (CSE) anesthesia. The patient was placed in the left side position and a disposable 16 gauge Toughy needle (portex) was introduced trough the L_2_/L_3 _epidural space under full aseptic condition without difficulty. The epidural space was located 5 cm from the skin via the loss of resistance technique with the midline approach. Then after 25 gauge 123 mm Whitacre spinal needle was inserted through the epidural needle, and free – flow of cerebrospinal fluid (CSF) was observed. Hyperbaric bupivacaine 0.5% 12.5 mg was injected over 30 sec without further CSF aspiration (speed of intrathecal injection approximately 0.05 ml.sec^-1^). Epidural catheter was inserted for the management of postoperative pain. The catheter was secured at 3 cm in the epidural space. Nothing was injected trough the catheter at this stage. The patient was kept in the left side position for 15 min.

The sensory block was evaluated by the pinprick test (22-gauge hypodermic needle), whereas motor block was evaluated by a modified Bromage scale (0 : no motor block; 1: hip block; 2: hip and knee block; 3 : hip, knee and ankle block). Ten minutes after the injection of local anesthetic solution, the sensory block level reached bilaterally to T_8 _dermatome level and the motor block level reched to Bromage 3 on both lower extremities.

Then, the patient was positioned supine on the operating table and surgery started at the 15^th ^min. In order to avoid the vasospasm of Reynaud's phenomenon; the temperature of the operating room was kept at 24°C and intra arterial cannula wasn't inserted.

Estimated blood loss during 70 min. surgery was minimal (approximately 250 ml). In addition to infused metilprednisolon 20 mg/six hours and regular insulin 8 IU/six hours, total fluids given during surgery was 2500 ml of 0.9% NaCl. There was no remarkable variation on hemodynamic and SpO_2 _values with respect to baseline values throughout the procedure.

After the spinal injection, the motor block was completely regressed at 195^th ^min and the sensory block was regressed at 225^th ^min. Because the patient appeared to be pain free in recovery room, nothing was given through the epidural catheter. The patient had been monitorized in post anesthetic care unit for 2 days, and catheter was removed on postoperative day 2. Then, she was transferred to the orthopedic ward. She was discharged from the hospital at postoperative day 14 uneventfully.

## Discussion

Although systemic sclerosis has been characterized as both localized and generalized forms, the spectrum of disease ranges from mild Reynolds's phenomenon to diffuse skin and visceral involvement [1]. Excessive accumulation of connective tissue components and structural vascular abnormalities have variable natural histories. Although most patients have long survival expectancy, minority of them progress to death because of the visceral involvement which contains dermal thickening, restrictive pulmonary disease, cutaneous calcification, congestive heart failure secondary to pulmonary hypertension, myocarditis, cardiac conduction abnormalities, diminished renal clearance of drugs, and lower esophageal sphincter dilatation. We know that all above conditions can contribute to many serious anesthesia related complications [3-5].

The clinical presentations of SSc and some anesthetic implications have been reviewed in a few articles[4,5]. And there are some published case reports on anesthesia techniques for the patients with SSc, but there isn't unique suggested technique for these patients. General anesthesia can lead to various problems such as difficulty of intubation and insertion of an iv cannula or measuring blood pressure. Moreover it was declared that performing a tracheotomy may be difficult because of sclerotic changes in the head and neck region [5].

As far as we know, there are three case reports in Medline about scleroderma. Both of them had preeclempsia and they were operated under general anesthesia. Unfortunately, one of them died because of pulmonary edema, pulmonary hypertension, sepsis and thrombocytopenia [6,7]. In another paper Bailey et al. reported that spinal anesthesia can be used for caesarean section in a patient with systemic sclerosis [8].

Regional anesthesia is frequently used in elderly patients undergoing surgery. Although the type of anesthesia has no substantional effect on peroperative morbidity and mortality, it makes sense that elderly patients would benefit from regional anesthesia because they remain awake throughout the procedures and obtain excellent postoperative pain control [9]. It should be kept in mind that, unpredictable spread and prolonged duration of action of local anesthetics may occur and regional anesthesia may be unacceptable for the patients with SSc[7,10]. In ' difficult to entubate ' cases despite the presence of high risk of spinal anesthesia some authors recommend regional anesthesia for patients with severe SSc [5,7]. In addition to this, it is widely believed that regional anesthesia has benefit for patients with severe pulmonary disease. Harald et al. reported that, even high thoracic regional anesthesia is well tolerated in patients with severe pulmonary disease, although FEV_1 _and vital capacity may be slightly decreased [11].

On the other hand, interstitial lung disease is reported to occur, in up to 80% of patients with scleroderma. Although, in the majority of the patients, the interstitial lung involvement is sub clinical and asymptomatic in the early stage; clinically significant interstitial lung disease is observed in approximately 40% of patients with systemic sclerosis and is a leading cause of morbidity and mortality [12].

Most experts rely on a combination of pulmonary functional tests for diagnosis of interstitial lung disease of SSc [12]. Pulmonary involvement is considered on the evidence of bibasal pulmonary fibrosis on chest radiogram or pulmonary functional tests (PFT) alteration (restrictive, obstructive or mixed pattern) on spirometry or less than 70% of predicted carbon monoxide diffusing capacity or the presence of all. Clinically significant restrictive lung disease is defined when an abnormal FVC with normal FEV_1_/FVC is observed [2]. In this patient, bibasal pulmonary fibrosis was seen on chest radiogram, and pulmonary functional tests (PFT) had alterations (restrictive or mixed pattern).

In our patient, because of the restricted oral aperture and decreased mandible – sternum distance and lung involvement we thought that regional anesthesia could be a better choice. Continuous spinal anesthesia techqnique (CSA) is recommended for diagnosis and treatment before peripheral sympathectomy in patients with secondary Reynoud's phenomenon and scleroderma [13]. So a carefully titrated continuous spinal anesthesia might have been chosen. However it was mentioned that the incidence of postoperative neurological deficits (such as headache, transient neurologic symptoms, cauda equina syndrome or meningitis) is significantly increased, following CSA technique [14,15] ; furthermore our patient needed only total hip arthroplasty and we were inexperienced on performing CSA technique. On account of these reasons combined spinal – epidural technique was chosen.

Preoperatively, drugs were administered to reduce the risk of aspiration and an ENT surgeon was available in the operating room for an urgent tracheotomy. In fact CSE technique was our first choice in this patient. Because of her flexion contractures and xifoscoliosis, it wouldn't have been performed. But we didn't face with any difficulty while performing CSE. It is known that, the difference between densities of cerebrospinal fluid and the solution injected has a major effect on intrathecal drug distribution [16]; for protecting the patient from the risk of bilaterally high spinal anesthesia, we used hyperbaric bupivacaine and kept the patient in the left side position after the spinal injection for 15 min. Generally, if the patient is kept in this position for 5 min. following injection, the block tends to be denser and achieve a high level on the operative side. But, in our patient, although the level of block was approximately at T_8 _level unexpectedly the block was bilateral. Although regional anesthesia may cause prolonged duration of action of local anesthetics in patients with scleroderma; in our patient the duration of local anesthetic was similar to McNamee's results. According to their results, when bupivacaine is used, duration of sensory block varies from 1.5 h to 4.6 h in patients without scleroderma [17].

## Conclusion

In respect of absence consensus and guidelines, the choice of regional or general anesthesia is unclear, this report describes the successful use of CSE technique in a patient with SSc and respiratory impairment. We concluded that the choice of anesthetic technique for patients with SSc should be made on an individualized basis, including an evaluation of the extent of the disease, technique and consideration of the surgical procedure, the circumstances of operating room and the choice of the patient.

## Competing interests

The authour(s) declare that they have no competing interests.

## Authors' contributions

GE, made substantial contributions to conception and design of case report, drafted the manuscript and revised it critically for important intellectual content. VT performed the anesthesia technique, drafted and revised the manuscript. FD initiated to write up the manuscript and had given the final approval of the version to be published. DÖ was involved in drafting and revising the case report along with first author. All authors read and approved the final manuscript.

## Pre-publication history

The pre-publication history for this paper can be accessed here:


